# A New Phosphorus Paradigm for the Baltic Proper

**DOI:** 10.1007/s13280-013-0441-3

**Published:** 2013-10-11

**Authors:** Anders Stigebrandt, Lars Rahm, Lena Viktorsson, Malin Ödalen, Per O. J. Hall, Bengt Liljebladh

**Affiliations:** 1 Department of Earth Sciences, University of Gothenburg, Box 460, 405 30 Göteborg, Sweden; 2 Department of Thematic Studies, Water and Environmental Studies, Linköping University, 581 83 Linköping, Sweden; 3 Department of Chemistry and Molecular Biology, Marine Chemistry, University of Gothenburg, 412 96 Göteborg, Sweden

**Keywords:** Internal load, Phosphorus, Baltic Sea, Mass balance model, Sediment, Eutrophication

## Abstract

The external phosphorus (P) loading has been halved, but the P content in the water column and the area of anoxic bottoms in Baltic proper has increased during the last 30 years. This can be explained by a temporary internal source of dissolved inorganic phosphorus (DIP) that is turned on when the water above the bottom sediment becomes anoxic. A load-response model, explaining the evolution from 1980 to 2005, suggests that the average specific DIP flux from anoxic bottoms in the Baltic proper is about 2.3 g P m^−2^ year^−1^. This is commensurable with fluxes estimated in situ from anoxic bottoms in the open Baltic proper and from hydrographic data in the deep part of Bornholm Basin. Oxygenation of anoxic bottoms, natural or manmade, may quickly turn off the internal P source from anoxic bottoms. This new P-paradigm should have far-reaching implications for abatement of eutrophication in the Baltic proper.

**Electronic supplementary material:**

The online version of this article (doi:10.1007/s13280-013-0441-3) contains supplementary material, which is available to authorized users.

## Introduction

Already in the first half of the twentieth century scientists showed that the phosphorus (P) content in eutrophic lakes was correlated to the oxygen condition in the bottom water (Mortimer 1941). The brackish Baltic Sea has been subjected to increased nutrient loads and resulting eutrophication since the 1950s. Despite regional attempts to curb the external N (nitrogen) and P loads the degradation of the ecosystem in the Baltic Sea continued with basin-wide Cyanobacteria blooms and increasing hypoxia. Reducing P loads has a crucial role to improve the conditions (Boesch et al. 2006). Recent research has shown a clear correlation between increasing extent of anoxic bottoms and an increase in phosphorus content of the Baltic Sea (Conley et al. 2002), suggesting an internal P source. This internal source has largely been explained by the increased deposition of organic matter and its subsequent degradation in sediments enhancing oxygen consumption and spreading of hypoxia–anoxia. Some of the resulting dissolved inorganic phosphorus (DIP) will under oxic conditions bind to iron oxyhydroxides. The latter will reductively dissolve in anoxic environments and mobilize DIP, enabling an increase in reloading of P to the water mass. This is according to the classical P-paradigm widely accepted after the first lake studies (Mortimer 1941).

Schindler (2012) reports a series of successful reversals of the cultural eutrophication process based solely on P load reduction in lakes, which are accompanied by a substantial time lag in their response. This lag is attributed to the return of a previous long-term increase in the sediment’s P stock, the internal P load. He also draws the conclusion that “estuaries with low-salinities, long water residence time, nitrogen-fixing Cyanobacteria and high denitrification will respond to phosphorous control much as lakes do”. Conley et al. (2009a) have shown in their review that even P leaking sediments are observed in the brackish Baltic Sea. This supports present focus on P dynamics in future Baltic proper remedy actions.

Iron-linked P flux is not the sole source of redox-dependent mobilized soluble reactive phosphorus (SRP). Remineralized inorganic poly-phosphate has been found to contribute to a minor part of the internal P efflux from sediments in a coastal inlet (Diaz et al. 2012). (Additional sources are further discussed in “A New P-Paradigm—Anoxic Bottoms as Temporal Net P Sources”.)

A first-order understanding of the large-scale turnover of P in the Baltic Sea is at hand if the estimated sources and sinks can close the P budget (mass balance). In the present paper where we first apply a P mass balance model to determine internal sources and sinks, and thereafter use available observations of benthic P fluxes to judge if the predicted internal source is in accordance with observations.

Reliable prediction of the response of the P content in a water body to changes of the P supply requires a proven dose–response model. Such a model must realistically describe major sources and sinks and their modes of operation. The total amount of P in the water column of the Baltic proper equals $V \cdot \bar{c}$, where *V* is the volume and $\bar{c}$ the spatial mean winter concentration that can be determined by vertical and horizontal integration of observed concentrations. The total sink is the sum of internal and external sinks, *Intsink* and *Extsink*, respectively. *Intsink* is due to burial in sediments within the Baltic Sea while *Extsink* is due to net exchange, i.e., export minus import to Kattegat. The overall P balance has been described by the following time-dependent budget (Wulff and Stigebrandt 1989),

1 $$ V\frac{{{\text{d}}\bar{c}}}{{{\text{d}}t}} = Extsource - Intsink - Extsink $$

**Fig. 1:**
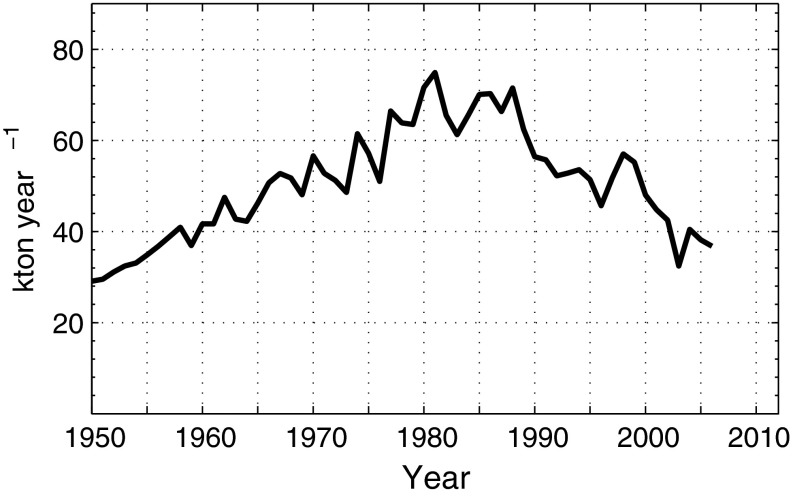
The annual external supply of total phosphorus to the Baltic Sea excluding Kattegat and the Belt Sea (after Gustafsson et al. 2012)

Both the rate of change of the total amount in the water column, $V\frac{{{\text{d}}\bar{c}}}{{{\text{d}}t}},$ and *Extsink* can be estimated from hydrographical data. Moreover, the sum of external sources, *Extsource*, is reasonably well known (Fig. 1). Within this formulation of the P balance, only *Intsink* is unknown in Eq. (1) and can thus be determined.

It is common to parameterize *Intsink* as the product of the specific internal sink, i.e., the sink per unit horizontal surface area (sediment burial), and *A*, the horizontal surface area of the basin that acts as a sink. The specific internal sink can be parameterized as the product of the winter P concentration in the surface layer, *c*, and the so-called apparent settling velocity *v* (m year^−1^) that should be independent of the magnitude of *c* (e.g., Wulff and Stigebrandt 1989). Thus,

2 $$ Intsink = c \cdot v \cdot A $$

**Fig. 2:**
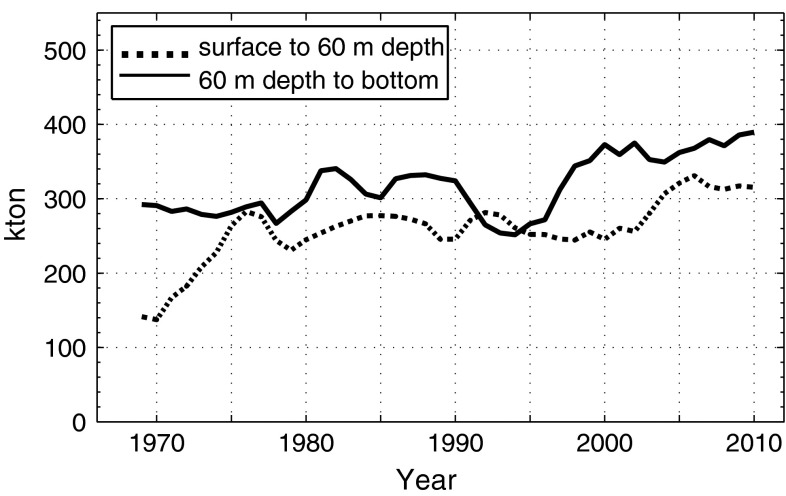
Phosphorus contents (Tot-P, winter data) above and below 60 m depth, respectively (3 years moving averages) (original data can be found in Electronic Supplementary Material)

This equation implies that a certain fraction of the annually produced particulate organic phosphorus (POP) is removed by the internal sink. Since *Extsink* is much smaller than *Extsource*, which is nowadays the case for the Baltic Sea as shown in “Results”, it follows from Eq. (1) that *Intsink* should be the dominating sink. It then follows from Eq. (2) that the winter concentration in the surface layer, *c*, should decrease when *Extsource* decreases. However, this has not happened in the Baltic proper where both *c*, and $\bar{c}$, have increased (Fig. 2) in spite of about 50 % reduction of *Extsource* since the 1980s (Fig. 1). Thus, the phosphorus model described by Eqs. (1) and (2) lacks one or more terms to correctly describe the P balance in the Baltic Sea.

**Fig. 3:**
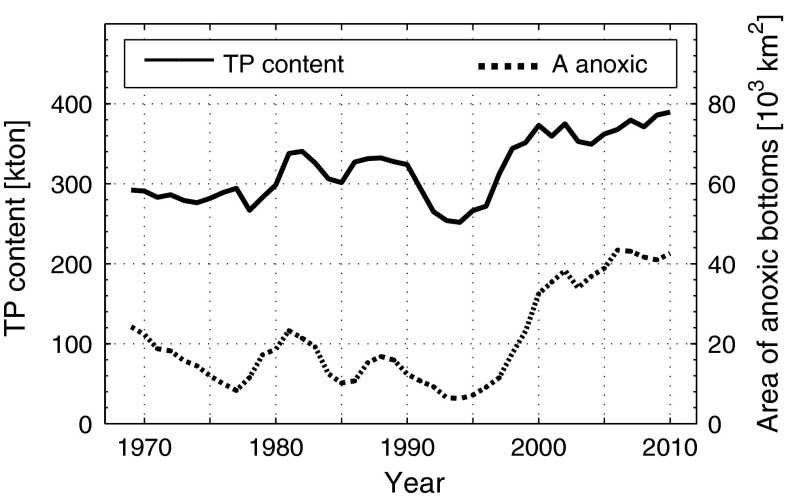
Content of total P (TP) below 60 m depth (from Fig. 2) and area of anoxic bottoms (*A* anoxic) in the Baltic proper (3 years moving averages) from 1968 to 2010 (anoxic bottom data from Hansson et al. 2011) (original data can be found in Electronic Supplementary Material)

Observations show that short-term changes of the phosphorus content in the deep water are positively correlated to the area of anoxic bottoms, see Fig. 3 and Conley et al. (2002). This has been explained as being due to variations of the sink capacity assuming that oxic bottoms are more efficient than anoxic bottoms as P sinks (Stigebrandt and Gustafsson 2007). However, it may also be explained by a variable source instead of a variable sink; anoxic bottoms may act as phosphorus sources that are turned on when the water above these bottoms become anoxic and turned off when the sediments are oxygenated. These two possible mechanisms of internal sinks and sources connected to anoxic bottoms are not included in the P model described above. In this paper we suggest that the missing source needed to balance the P budget is located on the anoxic bottoms. The introduction of internal net sources connected to anoxic bottoms in this paper’s P budgets is novel and will help understand how changes in internal sources influence eutrophication. The suggested change of focus from oxic bottoms as sinks to anoxic bottoms as sources may be regarded as a new P-paradigm for the Baltic proper, which is crucial for the understanding of how eutrophication may be diminished.

## Materials and Methods

**Fig. 4:**
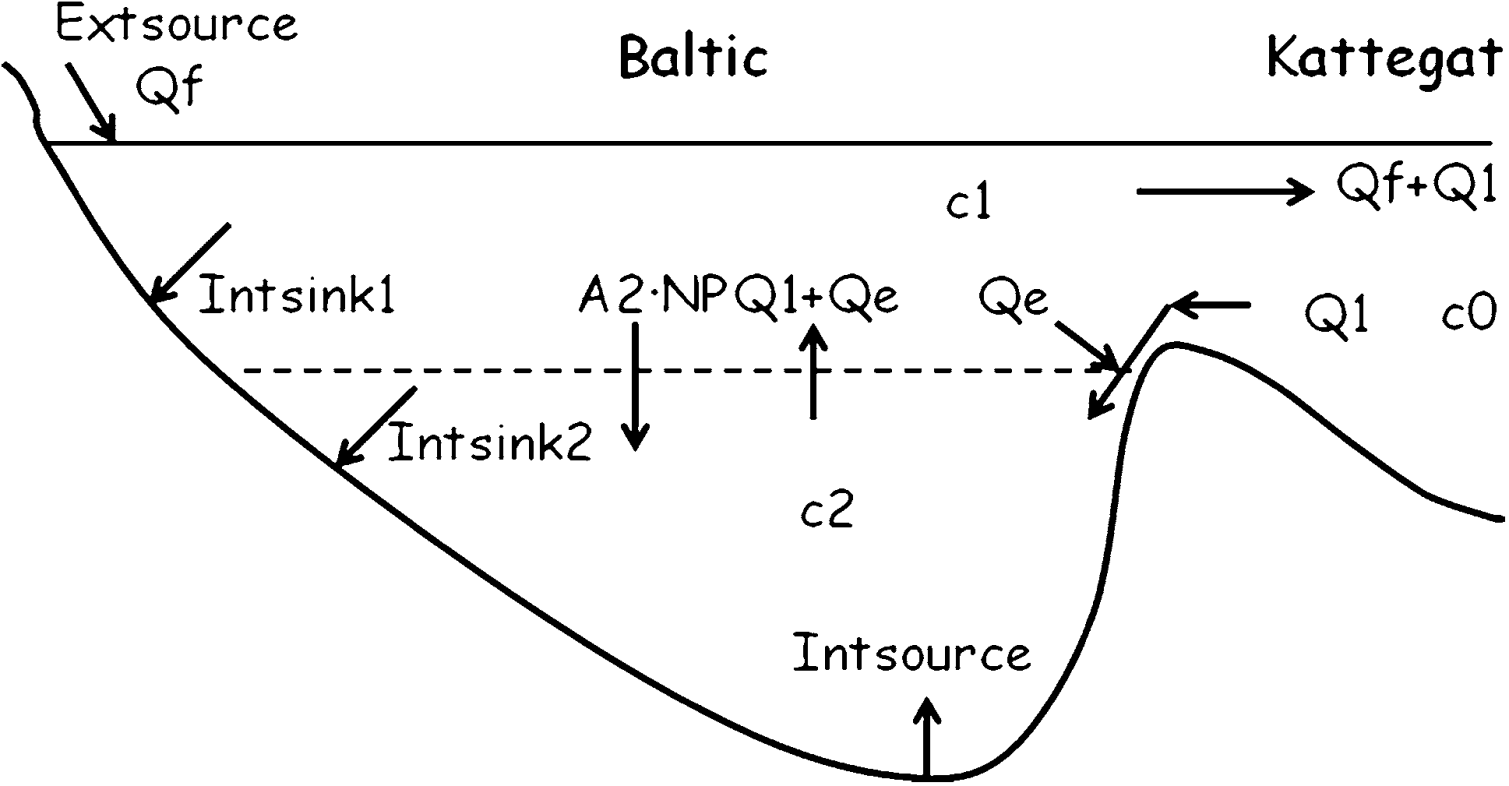
Phosphorus model of a two-layered Baltic Sea. The *stippled line* indicates the border between the upper and lower layers. *Q*_f_ is the freshwater supply; *Q*_1_ flow and *c*_0_ P-conc. of inflowing Kattegat water; *Extsource* and *Intsource* are external and internal sources; *Intsink1* and *Intsink2* are internal sinks; *Q*_e_ is entrained flow from upper to lower layer; *A*_2_ the horizontal surface area of interface between the upper and lower layers; NP new production of POP. *c*_1_ (*c*_2_) P-conc. of the upper (lower) layer

The phosphorus model of the Baltic Sea proposed here has the temporal resolution of 1 year. The water column is partitioned into an upper and a lower layer of volumes *V*_1_ and *V*_2_, and spatial mean winter concentrations $\overline{{c_{1} }}$ and $\overline{{c_{2} }}$, respectively (Fig. 4).

Most of the winter content of phosphorus in the upper 40 m is used for primary production. The specific vertical flux of POP on an annual basis, the so-called new production, is denoted NP (g P m^−2^ year^−1^). In deeper locations where the lower layer is present, POP is exported to this layer. The horizontal area of the interface between the upper and lower layers is *A*_2_ (m^2^). External sources, *Extsource* (g P year^−1^), contribute phosphorus to the upper layer via point sources and via freshwater runoff with a volume flow *Q*_f_ (m^3^ year^−1^). Denser water from Kattegat, volume flow *Q*_1_ (m^3^ year^−1^) and P-concentration *c*_0_, transports P to the lower layer. On its way towards the Baltic proper deep water, the dense Kattegat water entrains ambient water from the surface layer whereby the volume flow increases by *Q*_e_ (m^3^ year^−1^). Due to the inflow of denser water to the lower layer there is an equally large upward ‘return’ flow, *Q*_1_ + *Q*_e_, to the upper layer, carrying water of high DIP concentration. This occurs preferentially in autumn and winter when the turbulent surface layer is vertically well-mixed and entrains water from the lower layer. There are internal P sinks in sediments in both the upper and lower layers, *Intsink*_1_ and *Intsink*_2_, respectively. As supported by observations in “Phosphorus Flux from Bottom Sediments in the Bornholm Basin” and direct DIP sediment flux measurements (Viktorsson et al. 2013), it is assumed that only anoxic bottoms provide an internal source, *Intsource*, located in the lower layer. The net flux of P to Kattegat, *Extsink*, equals the outflow from the surface layer, $\left( {Q_{\rm f} + Q_{1} } \right)c_{1}$, minus the inflow to the lower layer, $Q_{1} \cdot c_{0}$. The phosphorus budget equations for the two layers are

3 $$ V_{1} \frac{{{\text{d}}\overline{{c_{1} }} }}{{{\text{d}}t}} = Extsource + \left( {Q_{1} + Q_{\text{e}} } \right)c_{2} - A_{2} NP - Q_{\text{e}} c_{1} - \left( {Q_{\text{f}} + Q_{1} } \right)c_{1} - Intsink_{1} $$

4 $$ V_{2} \frac{{{\text{d}}\overline{{c_{2} }} }}{{{\text{d}}t}} = - \left( {Q_{1} + Q_{\text{e}} } \right)c_{2} + A_{2} NP + Q_{1} c_{0} + Q_{\text{e}} c_{1} - Intsink_{2} + Intsource $$

By adding Eqs. (3) and (4), one obtains an equation for the long-term rate of change of the content of phosphorus of the whole system with volume *V* = *V*_1_ + *V*_2_ and spatial mean concentration $\bar{c}$, thus

5 $$ V\frac{{{\text{d}}\overline{c} }}{{{\text{d}}t}} = Extsource - Q_{\text{f}} c_{1} - Q_{1} (c_{1} - c_{0} ) - Intsink + Intsource $$

Here *Intsink* = *Intsink*_1_ + *Intsink*_2_. When adding the equations for the upper and lower layers the internal dynamics between the layers, e.g., the fluxes of dissolved and particulate matter (including bottom parallel fluxes of particulate matter), vanish. Only fluxes through, i.e., perpendicular to, external boundaries, including the bottoms, remain. Eq. (5) thus shows that the amount of P in the system tends to change by external sources, by the net exchange with Kattegat described by the two terms involving water fluxes (here called the barotropic transport *Q*_f_ and the baroclinic transport *Q*_1_, respectively), by the internal sink in sediments and by the internal source from anoxic sediments. Except for the term *Intsource*, Eq. (5) is identical to Eq. (1).

In “Estimation of the Internal P Source and Sink in the Baltic Proper”, the difference *Intsink* − *Intsource* will be determined by applying Eq. (5) to the Baltic proper at two different points of time when we know the other terms in Eq. (5). We then get two different values of this difference. To understand why the estimated difference *Intsink* − *Intsource* changes from one period to the other, we will couple internal sources to the area of anoxic bottoms.

In the application of the model in “Estimation of the Internal P Source and Sink in the Baltic Proper”, it is assumed that anoxic bottoms may act as internal P sources, *Intsource,* that is parameterized as follows

6 $$ Intsource = fs \cdot A_{\text{anox}} $$

Here *fs* (g P m^−2^ year^−1^) is the mean specific DIP flux from anoxic bottoms in the Baltic proper and *A*_anox_ is the area of such bottoms. In “Estimation of the Internal P Source and Sink in the Baltic Proper”, *fs* will be determined by applying Eq. (5) to the Baltic proper at two different points of time when the area of anoxic bottoms is known. This is to use the model in a diagnostic mode.

## Results

### Estimation of the Internal P Source and Sink in the Baltic Proper

We will apply Eq. (5) to the situation in 1980 and 2005, respectively, because they represent years with a small and large extent of anoxic area (Fig. 3) and a decreasing trend in external P load (Fig. 1). We then get two equations from which we can estimate the magnitude of the difference *Intsink* − *Intsource*. The total P content, increased from about 550 000 tons in 1980 to about 700 000 tons in 2010 (Fig. 2), i.e., the long-term mean of $V\frac{{{\text{d}}\bar{c}}}{{{\text{d}}t}}$, can be estimated to about 5000 tons P year^−1^. *Extsource* is shown in Fig. 1. The export of P is partitioned into two terms, the first, barotropic, term is the product of the freshwater run-off *Q*_f_ and the annual mean of the concentration $c_{1}$ in the surface layer, which we estimate to at most 0.5 mmol P m^−3^ in 1980 and 20 % higher in 2005; c.f. the change of P content in the upper layer (Fig. 2) in this time interval. The canonical value of *Q*_f_ equals 450 × 10^9^ m^3^ year^−1^. This gives a contribution from the barotropic term to the annual export of at most 7000 and 9000 tons P year^−1^ in 1980 and 2005, respectively. We neglect variations in export due to variations in *Q*_f_. The second, baroclinic, term depends on the product of the rate of inflow *Q*_1_ and the annual mean concentration difference $\left( {c_{1} - c_{0} } \right)$ between the surface water in the Baltic proper and the inflowing Kattegat water. This difference is probably close to zero, it may even be negative and if this happens, the baroclinic term turns into an import. The salinity balance of the Baltic proper shows that the long-term mean inflow is about equal to the rate of freshwater supply, i.e., *Q*_1_ ≈ *Q*_f_ (e.g., Stigebrandt 2001). For the time period considered, we estimate the net export of P to the Kattegat to be in the interval 5000 to 10 000 tons P year^−1^. This is in accordance to the estimated net flux, about 9500 tons P year^−1^, obtained by Rasmussen and Gustafsson (2003) based on a thorough analysis. The export in the 1970s was estimated to 5500 tons P year^−1^ (Wulff and Stigebrandt 1989).

For 1980 we estimate *Extsource* = 60 000, $V\frac{{{\text{d}}\bar{c}}}{{{\text{d}}t}}$ = 5000 and *Extsink* = 7000, thus

7 $$ Intsink-Intsource = 48\,000\,{\text{tons}}\,{\text{P}}\,{\text{year}}^{ - 1} . $$

For 2005 we estimate *Extsource* = 35 000, $V\frac{{{\text{d}}\bar{c}}}{{{\text{d}}t}}$ = 5000 and *Extsink* = 9000, thus

8 $$ Intsink-Intsource = 21\,000\,{\text{tons}}\,{\text{P}}\,{\text{year}}^{ - 1} . $$

The model thus shows that Eq. (5) is balanced if *Intsink* − *Intsource* decreases from 48 000 tons P year^−1^ in 1980 to 21 000 tons P year^−1^ in 2005, meaning that there is a net internal source in the Baltic Sea. This is the major result obtained from the model. However, to explain the changes observed in the Baltic Sea, we couple this result to the changing area of the anoxic bottoms in the Baltic proper. We study the case outlined in “Materials and Methods”, where anoxic bottoms act as net P sources and the sink is distributed over the total area. We choose to focus on this case, since it gives a specific P flux from anoxic bottoms (*fs*) that is commensurable with other independent estimates as shown below. Other cases are possible, e.g., only oxic bottoms act as sinks of P and no P is buried at anoxic bottoms; such a scenario is briefly discussed in “A New P-Paradigm—Anoxic Bottoms as Temporal Net P Sources”. To determine *Intsource* we use Eq. (6) with $A_{\text{anox}}$ equal to 20 000 and 40 000 km^2^, respectively, in 1980 and 2005 (Fig. 3, dashed line). For *Intsink* we use Eq. (2) with *c*(2005) = 1.2·*c*(1980), i.e., we assume that the winter surface concentration has increased by 20 % from 1980 to 2005 as described above. Using Eqs. (2) and (6) we rewrite Eqs. (7) and (8) and get the following equations for 1980 and 2005, respectively,

9 $$ c \cdot v \cdot A = fs \cdot 20\,000 + 48\,000 $$

10 $$ 1.2 \cdot c \cdot v \cdot A = fs \cdot 40\,000 + 21\,000. $$

Solving this equation system gives a 1980–2005 time averaged *fs* = 2.3 g P m^−2^ year^−1^. This assumes that *fs* has remained constant for the time period 1980–2005. The resulting *fs* implies that *Intsource* = 45 750 tons P year^−1^ in 1980 and *Intsource* = 91 500 tons P year^−1^ in 2005 (from Eq. 6). For *Intsink* we get from Eqs. (7) and (8) 93 750 tons P year^−1^ in 1980 and 112 500 tons P year^−1^ in 2005, respectively.

This model solution suggests that the internal source doubled in the period from 1980 to 2005 due to a doubling of the area covered with anoxic water. DIP released from deep anoxic bottoms is thus transported upwards by water circulation and, via production of POP in the surface layer, it may reach sinks at shallower areas. A similar mechanism, the P shuttle, does the same but is based on the redox-controlled Fe- and Mn-oxides’ ability to scavenge phosphate. This has been discussed and quantitatively estimated in the context of the Black Sea (Shaffer 1986) and recently applied to the Baltic proper pelagic redox-cline (Turnewitsch and Pohl 2010).

*Intsink* is an order of magnitude larger than *Extsink*. Some of the internal sink may be accounted for by export from the Baltic proper to the Bothnian Sea. It was estimated to be about 3900 tons P year^−1^ in the 1970s (Wulff and Stigebrandt 1989) and 5800 tons P year^−1^ in the 1980s (Wulff et al. 2001). Also the external P load to the Bothnian Bay, ca 8100 tons P year^−1^ in the 1980s (Wulff et al. 2001), should remain as sinks in this sea. These estimates show that about 85 % of our estimated internal sink in the Baltic Sea should be located within the Baltic proper, as sediment burial.

Application of our P model gives *fs* = 2.3 g P m^−2^ year^−1^ from anoxic bottoms and this can be compared to other estimates based on large-scale budgets. Conley et al. (2002) found that year-to-year increases of the phosphorus content in hypoxic water could be explained by sediment releases of on average 2 g P m^−2^ year^−1^ with a maximum release of 5 g P m^−2^ year^−1^ from hypoxic and anoxic bottoms. Similar fluxes of 2 g P m^−2^ year^−1^ from anoxic bottoms in the Eastern Gotland Basin (EGB) using the budget method described in Electronic Supplementary Material were estimated in Gustafsson and Stigebrandt (2007). The benthic DIP flux estimated with our P model can be validated by reliable direct measurements. By measurements in situ using benthic landers, DIP fluxes in the EGB of 4.2 ± 2.4 g P m^−2^ year^−1^ from bottoms overlain by anoxic water have been reported (Viktorsson et al. 2013). Estimates using hydrographical observations in the stagnant Bornholm Basin presented in “Phosphorus Flux from Bottom Sediments in the Bornholm Basin” show 3–5 times larger benthic DIP fluxes under anoxic than under oxic conditions. The in situ flux method and the basin budget method include both a possible source from anoxic bottoms and the reflux due to consumption of fresh organic matter. Therefore, they should give a larger flux than the flux (*fs*) from the model.

### Phosphorus Flux from Bottom Sediments in the Bornholm Basin

Using a budget method the oxidation rate of organic carbon, 28 g C m^−2^ year^−1^, was calculated by Stigebrandt and Kalén (2013) for the Bornholm Basin (BB) for the 1980s and later. Assuming that the organic matter is composed according to the so-called Redfield ratio, 0.7 g P m^−2^ year^−1^ should be remineralized to DIP. Consequently, the downward flux of P bound to POP is at least 0.7 g P m^−2^ year^−1^. According to these authors, oxygen consumption in the BB doubled from the 1960s and 1970s to the 1980s, meaning that NP in the 1960s was half of that in the 1980s. Please note that the budget method used by Stigebrandt and Kalén (2013) is insensitive to whether organic matter is entering directly from the surface layer or via lateral transport in the benthic boundary layers.

To calculate *FS*, the specific DIP flux from the sediments below 75 m, in the BB, a budget method (described in Electronic Supplementary Material) has been applied to the data set of total phosphorus from station BY5 (data available at www.smhi.se). The results for five decades, starting with the 1960s, are given in Table 1 where fluxes are presented separately for oxic and anoxic conditions in the water. Anoxic conditions culminated in the 1980s when the basin was anoxic about 20 % of the time. However, *FS* during anoxic conditions culminated in the 1990s when it reached the rate 8.6 g P m^−2^ year^−1^. The uncertainty of *FS* due to the uncertainty of the value of *the vertical diffusivity* is about ±20 % (see Table 1).

**Table 1:** Flux *FS* of DIP (g P m^−2^ year^−1^) from the bottom sediments beneath 75 m depth in the Bornholm Basin during oxic (*FS oxic*) and anoxic (*FS anoxic*) conditions during five decades. The percentage of the time the basin water has been oxic and anoxic is given as well as the number of estimates (No. estimates). The weighted averages (*FS aver*) for the basin accounts for the percentage of time that the basin water is oxic and anoxic, respectively

Decade	Oxic			Anoxic			*FS aver*
	*FS oxic*	% of time	No. estimates	*FS anoxic*	% of time	No. estimates	
1960s	0.8 ± 0.2	100	25	–	0	0	0.8
1970s	1.0 ± 0.2	96.7	25	3.0 ± 0.4	3.3	1	1.1
1980s	1.1 ± 0.3	79.7	16	3.8 ± 0.8	20.3	4	1.6
1990s	1.7 ± 0.2	88.1	43	8.6 ± 1.6	11.9	11	2.5
2000s	1.5 ± 0.3	86.1	60	6.5 ± 0.8	13.9	13	2.2

*FS* was typically 3–5 times greater during anoxic than during oxic conditions (Table 1). *FS* was larger than the downward flux of POP, as calculated from oxygen consumption above, both during oxic and anoxic conditions. It was twice the estimated downward flux of POP during oxic periods. In our analysis, conditions are defined as anoxic when the water is anoxic. However, DIP fluxes from the sediment should be more sensitive to whether or not the sediment–water interface is oxidized. This should prolong anoxic conditions since it takes time to oxidize the interface. This may explain why *FS* was larger than the downward POP flux also during oxic conditions in the water column. *FS* was more than 10 times larger than the downward flux of POP during anoxic conditions. This means that most of the DIP released under anoxic conditions must come from a storage that probably accumulated during earlier oxic periods. It is known that even the deepest parts of the BB were oxic and inhabited by, e.g., the long-lived bivalve *Macoma calcarea* in the first half of the twentieth century (e.g., Gerlach 1994). It is not known when this oxic period started but it ended between 1948 and 1956 as suggested by Gerlach (1994).

The specific flux of DIP from anoxic bottoms (*FS*) in the BB is greater than the specific flux averaged over all anoxic bottoms in the Baltic proper (*fs*) as estimated from the model in “Estimation of the Internal P Source and Sink in the Baltic Proper”. A contributing explanation to this, mentioned in “Estimation of the Internal P Source and Sink in the Baltic Proper”, may be that *FS* includes both the suggested internal source and the reflux of P from remineralization of the estimated supply of settling POP by NP of 0.7 g P m^−2^ year^−1^.

The last column in Table 1 gives the weighted decadal average of DIP flux from the sediments below 75 m depth (*FS aver*). Using this, the DIP flux in the 1960s was about 0.8 g P m^−2^ year^−1^. With *A*(75) = 5000 km^2^, the annual upward flux of DIP from the bottoms below 75 m was about 4000 tons P year^−1^ for this decade. It then increased and was about 11 000 tons P year^−1^ in the 2000s. The estimated supply of P by settling organic matter (POP) is only about 1800 and 3500 tons P year^−1^, respectively, for the two decades, as estimated above from the oxygen consumption. Thus, the deeper parts of BB appear to have acted as an internal source of about 2200 and 7500 tons P year^−1^ for the two decades, respectively. The accumulated net loss of P (upward DIP minus downward POP) from the deep bottoms in BB since 1960 should be about 50 g P m^−2^ (i.e., on average 1 g P m^−2^ year^−1^). This is less than the value of *fs* from the model for the Baltic proper, which is expected because, after all, the BB has been oxic most of the time.

## Discussion

### A New P-Paradigm—Anoxic Bottoms as Temporal Net P Sources

The model analysis in “Estimation of the Internal P Source and Sink in the Baltic Proper” shows that the evolution of the P content in the Baltic proper from 1980 to 2005 can be explained by an internal P source that varies with the area of anoxic bottoms. The main result of the modeling in “Estimation of the Internal P Source and Sink in the Baltic Proper” is that anoxic sediments in the Baltic proper act as sources with a mean specific DIP flux equal to 2.3 g P m^−2^ year^−1^. Observations from the BB show that the flux of DIP is a factor 3–5 greater when the bottom is covered by anoxic water than when it is covered by oxic water (“Phosphorus Flux from Bottom Sediments in the Bornholm Basin”). An average DIP flux from anoxic sediments in the Baltic proper of 4.2 g P m^−2^ year^−1^ was measured in situ using benthic chamber landers (Viktorsson et al. 2013), while estimates using Fick’s first law and pore water concentration gradients are found in the range 0.1–5.6 g P m^−2^ year^−1^ (Bolalek 1992; Hille et al. 2005; Jilbert et al. 2011; Mort et al. 2010). Still, we have one problem with this result, as mentioned in “Phosphorus Flux from Bottom Sediments in the Bornholm Basin”, and that is to explain why anoxic bottoms can act as substantial net sources under long periods. We are probably facing a transient state situation where P accumulated under oxic conditions in the sediments is released as DIP to the water column at a rate greater than the simultaneous supply to sediments. This is discussed below.

The oxygen consumption in the BB doubled from the 1960s to the 1980s (Stigebrandt and Kalén 2013). This is in general accordance with the increase of the winter surface water concentration of P in the Baltic Sea (Gustafsson et al. 2012). This supports the idea that the bottoms in most of the Baltic proper were oxic for a long time ending in the 1950s as discussed in “Phosphorus Flux from Bottom Sediments in the Bornholm Basin”. During this period, P might have accumulated in the bottoms due to burial of P in organic matter, accumulation of P associated with Fe-oxides, P accumulated in bacteria (as poly-phosphate) (Gächter and Meyer 1993; Mortimer 1941), or retention of another Fe(III)-P phase (Hyacinthe and Van Cappellen 2004; Lehtoranta et al. 2009).

When sediments turn anoxic, P previously retained in the oxidized sediments is released to the pore water as DIP. Adsorption of phosphate to iron oxides will both act to decrease the flux of P when the bottom water and the upper sediment is oxic, as well as increase the P flux when the sediment turns anoxic (Mortimer 1941). Another less discussed redox-dependent mechanism is storage of polyphosphates in bacteria under oxic conditions (Gächter et al. 1988; Sannigrahi and Ingall 2005; Goldhammer et al. 2010). This could possibly have the same or a larger influence on the benthic P flux in environments with changing redox conditions than iron adsorption (Jilbert et al. 2011). It has also been shown that sulfur bacteria use intracellularly accumulated polyphosphates as an energy source during anoxic conditions (Sannigrahi and Ingall 2005; Goldhammer et al. 2010).

At an oxic–anoxic turnover the DIP flux from sediment can therefore be much higher than what is expected from the degradation rate of organic matter. This release is most important in an environment with shifting redox conditions, since it will come to a halt when all easily reducible iron has been dissolved (Sundby et al. 1986) and poly-phosphates consumed. These release mechanisms can only explain the initial DIP release from bottoms when they become anoxic. However, much of the anoxic bottoms in the Baltic Sea have been anoxic for many years, and the model in “Estimation of the Internal P Source and Sink in the Baltic Proper” and the analysis of fluxes in the BB in “Phosphorus Flux from Bottom Sediments in the Bornholm Basin” show that also sediments that are anoxic for several years have a net release of P. Preferential regeneration of P from organic matter is supported from both flux measurements in long-term anoxic sediments (Ingall and Jahnke 1997; Viktorsson et al. 2012, 2013) and in sediment records (Slomp et al. 2002), and it means that P is recycled faster in anoxic sediments in relation to C than the Redfield C:P ratio of 106:1 would predict (Ingall et al. 1993). A mechanism explaining the preferential regeneration of P is the high (about 400:1) C:P ratio of microbial biomass, which can contribute to the high regeneration rate of P in relation to C in anoxic environments (Steenbergh et al. 2013). However, preferential mineralization of P cannot explain that sediments, which are anoxic for several years, are net sources of P, i.e., release more P than what is deposited on the sediment surface.

Hyacinthe and Van Cappellen (2004) provide strong evidence for the existence of an authigenic hydrous ferric phosphate mineral, possibly tinticite (Fe_4_(PO_4_)_3_(OH)_3_·5H_2_O), in estuarine sediments. It is suggested to form especially in the fresh but also in the brackish water part of the estuary, and to undergo only slow reductive dissolution in the anoxic part of the sediment due to adsorption of Fe(II) on the surface of the mineral. Lehtoranta et al. (2009) suggest that a similar Fe(III)-phosphate phase may accumulate in oxic sediments and be buried in underlying anoxic sediment also in brackish systems like the Baltic under non-eutrophic conditions. We find that one plausible, although somewhat speculative, explanation for the net release of P from long-term anoxic Baltic bottoms to be formation of such a hydrous ferric phosphate mineral in oxic Baltic proper sediments before the Baltic became eutrophic. During this period Fe(III) reduction was likely the dominant benthic respiratory pathway in the Baltic, and the hydrous ferric phosphate mineral could survive long-term also in anoxic sediment due to the scarce abundance of sulfide. Starting in the early 1960s, the Baltic developed into a eutrophic system, anoxia was spreading, and Fe(III) reduction was replaced by sulfate reduction as the dominant pathway for oxidation of organic matter in sediments (Lehtoranta et al. 2009). With the increasing concentrations of sulfide in sediments, the hydrous ferric phosphate mineral started to undergo reductive dissolution, releasing P to the bottom water, but slow enough due to the adsorbed Fe(II) and gradual increase of sulfide, so that the P release could take place during several years and possibly decades.

The net loss of P, through fluxes of DIP minus NP, from the BB was estimated over 50 years in “Phosphorus Flux from Bottom Sediments in the Bornholm Basin” and the total specific flux for this period became about 50 g P m^−2^. This requires a pool of P that can be mobilized over these years under varying redox conditions. In the BB a typical concentration of Org-P in the top 30 cm sediments is about 15 μmol g^−1^ (Mort et al. 2010). Assume an average water-content of 80 %. Then 20 % are solid particles. With an assumed density of about 2.65 g cm^−3^, the thickness Z of the sediment layer containing 50 g P m^−2^ becomes,

$$ Z = \frac{{50 \times 10^{ - 4} \,{\text{g}}\,{\text{cm}}^{ - 2} }}{{15 \times 10^{ - 6} \,{\text{mol}}\,{\text{g}}^{ - 1} \times 30.97\,{\text{g}}\,{\text{mol}}^{ - 1} \times 0.20 \times 2.65\,{\text{g}}\,{\text{cm}}^{ - 3} }} \approx 20\,{\text{cm}}. $$

This suggests that there is enough Org-P in the upper 20 cm of the sediment to support the estimated long-term efflux. Whether or not the stored phosphorus is labile is another question. In addition to the Org-P, there may be hydrous ferric phosphate mineral in the sediment undergoing dissolution and contributing to the long-term P efflux as proposed above. It should also be noted that our estimates are based on a sediment stock that has already supported a net loss of 50 g P m^−2^.

## Conclusion

There is an obvious correlation between the phosphorus content of the deep water and the area of anoxic bottoms as shown in Fig. 3 and in Conley et al. (2002). A large-scale P model including an internal P source from anoxic bottoms suggests that anoxia not only causes an initial DIP release from previously oxic sediments, but also turns on an internal phosphorus source, which in the Baltic proper equals about 2.3 g P m^−2^ year^−1^. Published data from different parts of the Baltic Sea over the last decades also show higher fluxes from anoxic than from oxic bottoms, and fluxes measured in situ from anoxic bottoms compare well with the model results (Viktorsson et al. 2013). An investigation of P fluxes in the BB using hydrographic data from 1960 to present (Table 1) shows that P fluxes from anoxic bottoms are 3–5 times greater than from the same bottoms under oxygenated bottom water conditions. We find that the idea of an internal P source that is turned on when bottom water becomes anoxic and turned off when sediments are oxygenated is well supported by evidence put forward in the present paper. However, we have only provided speculative explanations why anoxic bottoms in the Baltic proper may act as net P sources during periods as long as decades. Though the amounts of Org-P stored in the deep sediments of the BB, together with a possible storage of a hydrous ferric phosphate mineral, are probably sufficient to balance the estimated specific efflux of DIP from these sediments over five decades, we still cannot formulate a long-term dynamic P budget for these sediments. This is left to future research.

According to our estimates, the internal P source from anoxic bottoms in the Baltic proper was less than the external sources up to about 1995. In 2005, however, the internal source was almost three times larger than the actual external sources. From 1980 to 2005, the sum of external and internal sources increased by 20 750 tons P year^−1^ which explains why the phosphorus content in the Baltic increased steadily by about 5000 tons year^−1^ during that period. This in turn means that the export production (NP) from the upper layers has increased and thereby the areal extension of anoxia.

The only way for Nature to break the increasing eutrophication due to the positive feedback between eutrophication and anoxia is through oxygenation of anoxic bottoms. Anoxic bottoms need to be kept oxic for a long time (there are no estimates of this time so far) to obtain full effect of stopping the internal source and blocking the positive feedback between anoxia and eutrophication. An exceptional oxygenation took place in the 1980s and ended in the beginning of the 1990s, due to a weakening of the stratification in the Baltic proper. This included a lowering of the top of the halocline to 100 m in the EGB enabling an oxygenation of deeper, previously anoxic, bottoms (e.g., Stigebrandt and Gustafsson 2007). Much of the anoxia was removed and the phosphorus content decreased in the water column as shown in Fig. 3. However, anoxia expanded when the oxygenation event had passed, probably because the external source at that time was still large. A future oxygenation event of similar character that decreases the internal source by decreasing the area of anoxic bottoms might be more successful, because the external sources have decreased. However, nobody knows when such an exceptional natural oxygenation event may occur next time. Therefore, using manmade oxygenation might be considered to kick the Baltic proper into a less eutrophic state (Stigebrandt and Gustafsson 2007). If the external P-supply is sufficiently small, man-made oxygenation needs only to work for a limited time and may be termed restoration. Our computations of DIP leakage from the bottoms of the deeper parts of the BB clearly demonstrate that natural oxygenation radically decreases DIP leakage from these bottoms. By man-made oxygenation of the BB, deep-water anoxia would be eliminated (Stigebrandt and Kalén 2013), which should lead to reduced DIP leakage from the basin. The “excess” internal loading to the Baltic proper from the deep water in the BB due to anoxia was in the present paper estimated to be 7500 tons P year^−1^. This might be eliminated by continuous oxygenation of the deep water of the BB. Man-made oxygenation is not without controversy; see, e.g., comments by Conley (2012). However, several of the assumed negative effects, e.g., the viewpoint by Conley et al. (2009b), apply to both natural and man-made oxygenation. Rigorous analyses of consequences have to be done before man-made oxygenation may be recommended.

## Electronic supplementary material

Below is the link to the electronic supplementary material.

Supplementary material 1 (PDF 165 kb)
