# Supplementary material for: A New Phosphorus Paradigm for the Baltic Proper
Source: Ambio. 2013 Oct 11;43(5):634–43. doi: 10.1007/s13280-013-0441-3 (PMC4132467; doi:10.1007/s13280-013-0441-3)
Supplement: Supplementary file 1 — Supplementary material 1 (PDF 165 kb) [file 13280_2013_441_MOESM_ESM.pdf]

AMBIO

Electronic Supplementary Material

A New Phosphorus Paradigm for the Baltic Proper

Anders Stigebrandt, Lars Rahm, Lena Viktorsson, Malin Ödalen, Per O.J. Hall, Bengt Liljebladh

S1. Supply of P, the P-content above and below 60 m depth and the area of anoxic bottoms in the Baltic proper

The second column gives the annual supply of Phosphorus (P) to the Baltic Sea inside the Sound and the Belts (after Gustafsson et al., 2011). The third and fourth columns show the P content above and beneath 60 m depth, respectively. Data from the main basins in the Baltic proper have been used together with the hypsographic functions of the basins. The fifth column gives the area of anoxic bottoms in the Baltic proper (after Hansson et al., 2011).

Year	TotP supply to Baltic [kton year^{-1}]	TotP content surface to 60 m [kton]	TotP content 60 m to bottom [kton]	Area of anoxic bottoms [10^3 km^2]
1950	29.1			
1951	29.5			
1952	31.1			
1953	32.4			
1954	33.1			
1955	34.9			
1956	36.7			
1957	38.8			
1958	40.9			
1959	36.9			
1960	41.7			
1961	41.7			
1962	47.5			
1963	42.7			
1964	42.2			
1965	46.3			
1966	50.7			
1967	52.8			
1968	51.8	147.7	293.5	24.1
1969	48.1	106.6	278.9	35.3
1970	56.6	170	304.2	13.3
1971	52.8	135.4	289.4	18.5
1972	51.2	195.6	255.3	24.6
1973	48.6	216.1	314	11.6
1974	61.5	213	267.8	11.3
1975	57.2	254.6	246.9	20.5
1976	51	323.2	330.4	4.1
1977	66.5	271.1	290	5.4
1978	63.9	233	262.4	15.4
1979	63.5	227.7	247.6	13.8
1980	71.6	231.6	339.1	22.5
1981	74.9	275.5	308.7	19.6
1982	65.5	254.8	365.4	27.8
1983	61.2	258.1	347.4	16.8
1984	65.5	297.8	265.2	13
1985	70.1	275.4	305.9	7.7

1986	70.3	258.7	333.1	9.9
1987	66.3	295.1	341.8	14.5
1988	71.5	264.2	319.2	21.4
1989	62.5	239.3	335.6	14.6
1990	56.4	232.7	327.5	11.7
1991	55.8	264.9	309	11
1992	52.2	315.9	246.3	9.3
1993	52.8	263.7	239.6	7.4
1994	53.6	255	275.9	2.8
1995	51.4	263.8	239.6	8.7
1996	45.7	237.6	283.8	10
1997	51.8	254.2	292.3	8.7
1998	57	245.9	361.1	15.9
1999	55.2	232.9	379.1	28.3
2000	48	287.4	313.7	25.1
2001	44.8	215.8	426.1	44
2002	42.5	277.7	338.2	37.1
2003	32.4	274.8	360.7	33.8
2004	40.5	288.4	359.3	31.1
2005	38.2	357.1	328	45.8
2006	36.8	317.7	399.5	39.8
2007		318.3	375.9	44.7
2008		312.8	363	44.9
2009		306.5	374.4	35.4
2010		332.5	419.7	42.6
2011		307.3	374.5	49.7

References

Gustafsson, B.G., F. Schenk, T. Bleckner, K. Eilola, H.E.M. Meier, B. Muller-Karulis, T. Neumann, T. Ruhoho-Airola, et al. 2012. Reconstructing the Developmenmt of Baltic Sea Eutrophication 1850-2006. Ambio 41 (6):534-548. DOI: 10.1007/s13280-012-0318-x

Hansson, M., L. Andersson and P. Axe. 2011. Areal Extent and Volume of Anoxia and Hypoxia in the Baltic Sea, 1960-2011. SMHI Swedish Meteorological and Hydroplogical Institute, Report Oceanography No. 42, Norrköping, Sweden, 76 pp.

S2. Method to estimate phosphorus fluxes during stagnant periods from hydrographical data

In section 3.2 we estimate *FS*, the specific flux of DIP (g P m^{-2} s^{-1}) from sediments below 75 m depth in the 100 m deep Bornholm Basin. The flux from these sediments has been estimated for so-called stagnant periods, i.e. periods lacking significant inflow of new denser deep water in the period between two consecutive hydrographical observations. Here we use observations at 70 and 80 m depth. The flux was estimated as follows

$$FS = \frac{V(75)}{A(75)}\frac{dP(80)}{dt} + F(75) \qquad (S1)$$

Here *V(75)* is the volume beneath and *A(75)* is the horizontal area at 75 m depth which is used as a proxy for the area of the bottom beneath this depth. The first term on the right hand side is the rate of change of the storage of P per unit area which is proportional to the observed concentration change $dP = P_{n+1}(80) - P_n(80)$ at 80 m depth during the time interval $dt = T_{n+1} - T_n$ where T_{n+1} and T_n are the times when profiles number n+1 and n were taken. The second term, *F(75)*, is the turbulent diffusive flux through the 75 m level given by *Eq. (S2)* below. When computing *FS* for the time between $t=T_n$ and $t=T_{n+1}$ the average of *F(75)* of these two times is used.

The diffusive flux $F_n(75)$ of DIP through the 75 m depth level time at $t=T_n$ is computed (Fick's First Law), as follows

$$F_n(75) = \kappa_n(75)\,\frac{P_n(80) - P_n(70)}{\Delta z} \qquad (S2)$$

Where Δz =10m and $P_n(80)$ and $P_n(70)$ are observations at 80 and 70 m depth, respectively. The horizontal mean vertical turbulent diffusivity $\kappa(z)$ varies with depth z and with the vertical stratification, here given by the buoyancy frequency *N* defined by *Eq. (S4)*. For 75 m depth, κ is defined by

$$\kappa_n(75) = \frac{a(75)}{N_n(75)} \qquad (S3)$$

Here *a(75)* is an empirical "intensity" coefficient of the turbulence, that equals $(1.3\pm0.9)\cdot10^{-7}$ (m^2 s^{-2}) for the Bornholm Basin (Stigebrandt and Kalén 2012). This is close to values used in oceanographic circulation models applied to the Baltic Sea, (e.g. Eilola et al. 2011). The buoyancy frequency at 75 m at time $t=T_n$, $N_n(75)$, is computed as follows from the observed density $\rho(70)$ and $\rho(80)$ at 70 and 80 m depth, respectively,

$$(N_n(75))^2 = \frac{g}{\rho_0}\frac{\rho_n(80) - \rho_n(70)}{\Delta z} \qquad (S4)$$

Here ρ_0 is a reference density and *g* the acceleration of gravity.

References

Eilola, K., B.G. Gustafsson, I. Kuznetsov, H.E.M. Meier, T. Neumann and O.P. Savchuk. 2011. Evaluation of biogeochemical cycles in an ensemble of three state-of-the-art numerical models of the Baltic Sea. Journal of Marine Systems 88 (2): 267-284. DOI: 10.1016/j.jmarsys.2011.05.004

Stigebrandt, A. and O. Kalén. 2013. Improving Oxygen Conditions in the Deeper Parts of Bornholm Sea by Pumped Injection of Winter Water. Ambio, 42 (5): 587-595. DOI:10.1007/s13280-012-0356-4
